# On the number of founding germ cells in humans

**DOI:** 10.1186/1742-4682-2-32

**Published:** 2005-08-24

**Authors:** Chang-Jiang Zheng, E Georg Luebeck, Breck Byers, Suresh H Moolgavkar

**Affiliations:** 1 Department of Occupational and Environmental Medicine, Regions Hospital, University of Minnesota, 640 Jackson Street, Saint Paul, MN 55101, USA; 2 Division of Public Health Sciences Fred Hutchinson Cancer Research Center, 1100 Fairview Avenue North, Seattle, WA 98109, USA; 3 Department of Genome Sciences, University of Washington, Seattle, WA 98195, USA

## Abstract

**Background:**

The number of founding germ cells (FGCs) in mammals is of fundamental significance to the fidelity of gene transmission between generations, but estimates from various methods vary widely. In this paper we obtain a new estimate for the value in humans by using a mathematical model of germ cell development that depends on available oocyte counts for adult women.

**Results:**

The germline-development model derives from the assumption that oogonial proliferation in the embryonic stage starts with *a *founding cells at *t *= 0 and that the subsequent proliferation can be defined as a simple stochastic birth process. It follows that the population size *X*(*t*) at the end of germline expansion (around the 5^th ^month of pregnancy in humans; *t *= 0.42 years) is a random variable with a negative binomial distribution. A formula based on the expectation and variance of this random variable yields a moment-based estimate of *a *that is insensitive to the progressive reduction in oocyte numbers due to their utilization and apoptosis at later stages of life. In addition, we describe an algorithm for computing the maximum likelihood estimation of the FGC population size (*a*), as well as the rates of oogonial division and loss to apoptosis. Utilizing both of these approaches to evaluate available oocyte-counting data, we have obtained an estimate of *a *= 2 – 3 for *Homo sapiens*.

**Conclusion:**

The estimated number of founding germ cells in humans corresponds well with values previously derived from chimerical or mosaic mouse data. These findings suggest that the large variation in oocyte numbers between individual women is consistent with a smaller founding germ cell population size than has been estimated by cytological analyses.

## 1. Introduction

Despite great strides in our understanding of the genetic regulation of germ cell determination in recent years [1], the size of the founding germ cell population in humans remains obscure. Due to this uncertainty, it is difficult in a clinical environment to estimate the probability that a mutant allele known to be mosaic in the somatic tissues of a parent will be transmitted to offspring. Even in the mouse, where experimental approaches are feasible, the number of founding germ cells (FGCs) has proven difficult to establish. Cytochemical methods have suggested FGC numbers varying from 45 cells [2] to 193 cells [3]. On the other hand, genetic analysis of artificially generated chimerical cellular populations in the mouse indicate that there are only 2 to 9 cells that actually contribute to the germ cell population [4-10]. In this communication, we derive a new method that is applicable to both humans and laboratory animals. This approach exploits a "founder effect" phenomenon that has previously been shown to be amenable to mathematical analysis [11,12]. Specifically, such analysis has shown that, if a population is descended from a small set of ancestral founders, the population size should exhibit substantial variance. Due to exponential expansion of the germline from the small number of founding cells, modest variation of cell cycle parameters between individual founding cells would be amplified into substantially higher levels of variance at later stages of development. Using a stochastic model to reconstruct the germ-cell development in human females, we show that the large variance observed in counts of human oocytes is consistent with the initial origin of these cells from a much smaller FGC population than is often assumed.

## 2. Overview

Our approach enables us to derive an estimate of the initial FGC population size on the basis of reliable data describing the number of oocytes present at various later stages of human development, when ovarian tissue is more readily available for analysis. Cytological counts of oocytes have shown not only that the size of the female germ cell population varies substantially between individuals of the same age, but also that its age-dependent magnitude is biphasic [13]. Numbers of germ cells increase during the first half of fetal development and then begin a progressive decline that extends throughout the reproductive years. The initial phase initiates with the separation of the germline from the soma, probably taking place no later than the peri-implantation stage (about 9 days after fertilization) [13,14]. Following this initial establishment, mitotically active oogonia undergo an exponential increase in number while only a small fraction of them show any sign of degeneration. At about five months of fetal development (5/12 = 0.42 year), the population reaches its peak as the oogonia enter into meiotic arrest. The germ cells (now defined as primary oocytes) become invested by layers of nurturing granulosa cells to form the follicles, which are readily recognized and enumerated by microscopic examination. The second phase of female germline development, spanning the period from the late embryonic stage to the onset of menopause in adult females (*t *= 0.42 – 52 years), is characterized by a progressive decline in the number of follicles, largely due to apoptosis [15]. This decline is approximately exponential, but is accelerated in women older than age 38 [16]. Among the million or so oocytes present late in fetal development of the mother, the vast majority will undergo apoptosis while only 300–400 will progress fully through maturation and undergo ovulation during the woman's reproductive life. Eventually, when the number of oocytes in the resting pool falls below 1000, menopause occurs [16].

## 3. Germ-Cell Kinetics

### Oogonium-Birth Model

**Figure 1:**
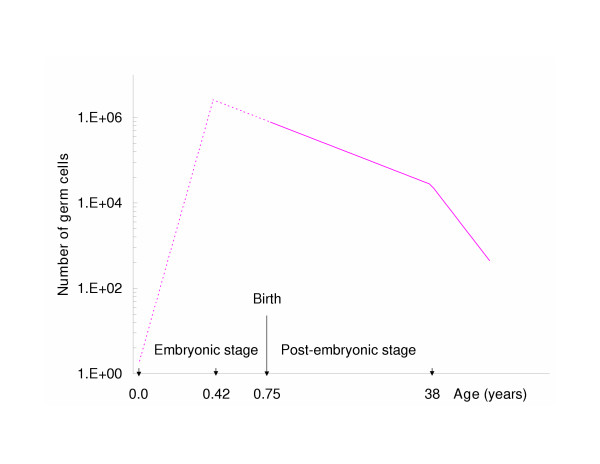
The number of female germ cells in humans undergoes three distinct rate changes, as diagrammed here and defined in the model. For the sake of clarity, the age coordinate is expanded artificially during the embryonic phase. The proliferative phase initiates at the time of germline-soma separation (*ca.* 9 days after fertilization; *t *= 0.0 year) and ends after 5 months of gestation (*t *= 0 – 0.42 year). The declining phases begin later in fetal life and continue into adulthood (*t *= 0.42 – 52 years) with an accelerated rate of oocyte depletion beginning at age 38 [16]. The dotted line shown during the embryonic stage emphasizes that oogonial cell counts from this period are inaccessible to reliable determination.



The stochastic model we use to describe germline development consists of two separate dynamic components (Figure 1). During the early embryonic stage (*t *= 0 – 0.42 year), a pure-birth model [11] can be used to describe the rapid proliferation of oogonia. At time *t *= 0, the germline is founded by *a *ancestral cells (FGCs) that are newly separated from the soma. At time *t *(0 ≤ *t *≤ 0.42), the number *X*(*t*) of oogonia follows a negative-binomial probability distribution [11]:

Here *λ *is the oogonial division rate. The expectation and variance of the random variable *X*(*t*) are *E*[*X*(*t*)] = *ae*^*λt*^, *Var*[*X*(*t*)] = *ae*^*λt*^(*e*^*λt*^-1) ≈ *ae*^2*λt*^, respectively. Note that the approximation holds true if *e*^*λt *^is large (for humans, *ae*^*λ*·0.42 ^≥ 10^6^). The moment ratio *a *≈ *E*^2 ^[*X*(*t*)]/*Var*[*X*(*t*)] then yields an estimate of the number of FGCs.

If one were dependent on using the pure-birth model to estimate *a*, this could be accomplished by collecting gonadal tissues from a series of abortices and establishing the total number of oogonia in each specimen (*x_i_*(*t_i_*), *i *= 1,2,..., *I*) by microscopic evaluation. However, reliance on access to fetal tissue clearly has several drawbacks. First, many spontaneous abortions are associated with chromosomal aberrations [17] and may therefore display an abnormal pattern of growth kinetics. Second, access to non-diseased fetuses for research is limited by ethical concerns. And, third, microscopic examination of fetal tissues from an early stage of pregnancy is technically challenging. The boundaries of fetal gonad are not clearly demarcated from surrounding cell types and the oogonial cells are difficult to distinguish from the somatic cells. On the other hand, the ovarian follicles that arise at later stages of development are cytologically distinct and can be enumerated with precision. The following derivation of a pure-death model for germ cell dynamics enables us to use this more precise enumeration to advantage.

### Oocyte-Death Model

A pure-death model, as described by Bailey [11], can be used to obtain an explicit formulation for the declining phase of germ cell numbers after proliferation has ceased and the apoptotic decline has begun (*t *= 0.42 – 52 years). Consistent with the findings of Faddy et al. [16], we permit the rate of oocyte loss from the resting pool to vary with age *t*. The cumulative rate function *f*(*t*) is defined as follows:



Conditional on the initial number *X*(*t*) = *n *of oocytes at *t *= 0.42 year, the number of oocytes in the resting pool at age *t *(0.42 ≤ *t *≤ 52 years) now follows a binomial probability distribution [11]:



## 4. Estimation Methods

The unknown parameters (*a*, *λ*,*μ*_1_, *μ*_2_) can be estimated using two different methods. The moment-based method estimates only the number of FGCs (parameter *a*), while the maximum likelihood method estimates all four parameters (*a*, *λ*, *μ*_1_, *μ*_2_) simultaneously.

### Moment-Based Method

As mentioned above regarding the oogonium-birth model, the random variable *X*(*t*) follows a negative binomial distribution, and therefore the moment ratio *E*^2^[*X*(*t*)]/*Var*[*X*(*t*)] yields an estimate of *a*. This relationship holds true with oocyte depletion (oocyte-death model) following the period of exponential growth. As a verification, notice first that the probability-generating function for the negative binomial probability distribution at *t *= 0.42 is *P*_*X*(0.42)_(*s*) = {1 - *e*^*λ*·0.42^(1 - *s*^-1^)}^-*a*^. Therefore, the probability-generating function for the binomial probability distribution at *t *> 0.42 (conditional on *X *(0.42) = *n*) is *P*_*X*(*t*)|*X*(.42)_(*s*) = ((1 - *e*^- *f*(*t*)^) + *e*^- *f*(*t*)^*s*)^*n*^. The compounded probability-generating function (*t *> 0.42) is then given by *P*_X(*t*)_(*s*) = . The mean and variance for oocyte population size in adult women can be derived from the first and second derivatives of , .

### Maximum Likelihood Estimation (MLE)

Although the moment ratio is simple to compute, its derivation requires a large sample size and provides no estimates of the oogonium-birth rate (*λ*) and the oocyte-death rates (*μ*_1_, *μ*_2_). The maximum likelihood method is not subject to these difficulties. To derive the likelihood function, note first that Equation 3 (pure-death model) is a probability function conditional on Pr[*X*(0.42) = *n*] (Equation 1; pure-birth model). For each of the oocyte counts obtained from a series of autopsies (*x*_*i*_(*t*_*i*_), *i *= 1,2,..., *I*) during the post-embryonic stage (0.42 ≤ *t*_*i *_≤ 52 years), we can combine them to define:



Since each observation *x*_*i*_(*t*_*i*_) makes a contribution like *L*(*x*_*i *_| *a*, *λ*, *μ*_1_, *μ*_2_) to the likelihood, the final likelihood  of the entire data set is the product of all such terms (0.42 ≤ *t*_*i *_≤ 52 years), such that . To maximize , note that the term  is (for a fixed *x*_*i*_(*t*_*i*_) but variable *n*) a negative binomial up to a constant. Therefore, we use importance sampling [18] from negative binomials to evaluate the likelihood in Equation 4 numerically. In practice, we first generate samples of *n *from this distribution and then sum the values of the negative binomial probabilities for *a *given the sampled values of *n*. We obtain stable likelihood estimates with as few as 100 samples of *n*. During the process of searching the parameter space, we also restrict the parameter *a *to be a positive integer (*i.e*., *a *= 1,2,3,...).

## 5. Analysis of Oocyte-Counting Data

**Figure 2:**
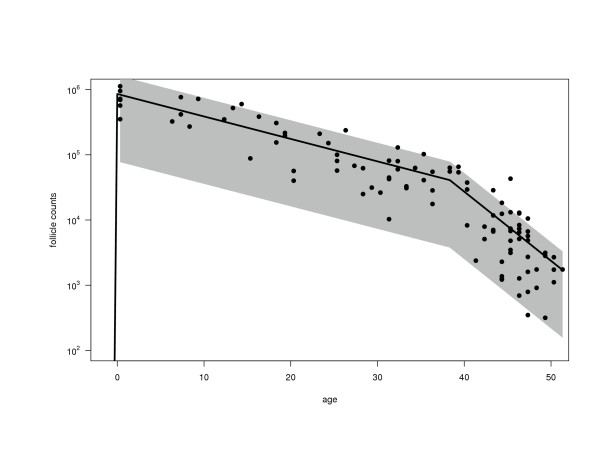
Shown in this diagram are published counts of follicles per individual [16,19] obtained by autopsy in adult stages, when each follicle contains a single oocyte. The data are analyzed with the compound birth-then-death model as described in the text. Observations with follicle counts < 100 were considered unreliable and were excluded from the analysis. The solid line represents the expected number of oocytes at each age in the postembryonic stages based on the model given these data. The shaded area is the pointwise 80% confidence interval. The MLEs of the parameters are:  = 2,  = 31.2/year,  = 0.079/year,  = 0.248/year.

Using the above algorithms, we have analyzed published oocyte counts from 102 human females [16,19]. These computations yield MLEs of the four parameters, which are:  = 2,  = 31.2/year (95% CI: 30.3 – 31.9),  = 0.079/year (0.067 – 0.090) and  = 0.248/year (0.204 – 0.283). The 95% confidence intervals (95% CI) are based on Markov chain Monte Carlo methods [20]. The expected number of oocytes (solid curve) and the pointwise 80% CI (shaded region) for the predicted counts on the basis of the model are shown together with oocyte counts in Figure 2.

The large MLE of *λ *justifies our use of the moment-based method to estimate the parameter *a*. To enable moment-based analysis we have grouped the data into 5 discrete age intervals [16] and calculated the mean and variance for each age interval. Computing the moment ratio index for each age interval yields the values shown (Table 1) and an overall average of  = 2.7. This value agrees well with the MLE and with those values ( = 2 – 9) that were derived from the segregation of genetic markers in the mouse [4-10].

**Table 1:** Oocyte-counting data from Faddy et al. [16].

Age	Sample Size	Mean	S.E.	Ratio Index
19–30 years	5	78980	15580	5.1
31–35 years	13	25300	4860	2.1
36–40 years	14	21450	2650	4.7
41–45 years	32	7320	1450	0.8
>45 years	36	1880	310	1.0

## 6. Discussion

Our estimated values of the founding germ cell number *a *(MLE  = 2, moment estimate  = 2.7) differ substantially from the estimates made in previous studies [2,3] that relied on cytochemical staining of embryonic material. This discrepancy might best be explained by assuming that not all cells sharing the same cytological phenotype (alkaline phosphatase staining) in common with a FGC population proceed through that course of development. This view is supported by the observation that alkaline phosphatase is present not only in germ cells of the mouse, but also in somatic cells that surround these germ cells [21]. Recent description of cytochemical markers that are more specifically restricted to the germ cells confirms that the expression of alkaline phosphatase occurs in a wider spectrum of cell types [22].

Genetic analysis provides a more stringent approach to germ cell enumeration in the mouse, where experimental crosses permit reliable determination of marker transmission. If a mutation or other stably transmissible cellular property arises early in development, only a subset of cells will display the mutation at later stages. Such *mosaic *presence of the trait provides an opportunity to help define the stage at which the germ cell precursors (FGFs) had become segregated from the predominant somatic cell population [23,24]. If the extent of mosaicism were closely similar between soma and germline, this would indicate that the size of the FGC population is large, since it would have provided a representative sample of the mosaicism originally present throughout the early embryo. To the contrary, if the correlation between somatic and germline mosaicism is weak, the number of FGCs must be limited and the transmission of any somatic markers to offspring should be more stochastic. This type of analysis [4-10] generally predicts small number of FGCs, consistent with our derivation from the stochastic modeling of oocyte counts.

The present statistical analysis indicates that germ cell proliferation occurring during embryogenesis is characterized both by a small FGC population ( = 2) and by a rapid rate of cell division ( = 31.2/year). Although we have estimated all four parameters of the model (*a*, *λ*, *μ*_1 _and *μ*_2_) in concert using the oocyte-counting data collected post-embryonically, the division rate *λ *can be verified independently by microscopic study of embryonic tissues. In a recent publication, Bendsen et al. [25] reported their evaluation of 10 fetal gonad specimens between the ages of 6 and 9 weeks. Using morphological clues to distinguish germ cells from somatic cells, these authors were able to establish the number of germ cells per tissue sample throughout this period. We used exponential regression against fetal age *t *to analyze the data of Bendsen et al. and obtained a direct estimate of  = 35.4/year (95% CI: 17.2/year – 53.7/year). Thus, our estimate of the division rate of the FGC is in good agreement with the experimental data.

It has recently been reported [26] that mammalian ovaries contain stem germ cells that are competent to enter into mitosis at a late stage of development. If confirmed by further work, this finding would necessitate some modification of the model proposed here. However, additional computation suggests that the potential effect on the estimate of *a *is likely to be small. To show this, we replace the pure death model for oocyte dynamics with a birth-and-death model. Also we assume that both the cell-death rate *μ *and the cell-birth rate *ν *(*μ *>*ν*) are constant during the post-embryonic stage (*t *> 0.42). The re-derived compounded probability generating function then becomes . From this, we obtain the expectation (*E*[*X*(*t*)] = *ae*^*λ*.0.42-(*μ-ν*)*t*^) and the variance of the oocyte population. Numerical evaluations of *Var*[*X *(*t*)] using a range of parameter values suggest that the variance and the estimate of *a *are largely determined by the observed difference between *μ *and *ν *(*Var*[*X*(*t*)] ≈ *ae*^2(*λ*·0.42-(*μ-ν*)*t*)^). In other words, our conclusion will change very little as long as the cell-death rate *μ *is substantially larger than the cell-birth rate *ν *(even if *ν *> 0). Because the observation on continued proliferation of germ cells in the adult female is new [26] and additional evidence will need to be collected, a fuller discussion of the modeling issues is deferred here.

The validity of our model clearly relies on certain assumptions that might be refuted by future analyses of tissue dynamics. Specifically, we have assumed that growth of the germ cell population is exponential and involves no significant cell death during gestation and also that the subsequent apoptotic decline is exponential. Furthermore, we have assumed that the sub-populations derived from each individual FGC grow and decline independently of one another. In addition, the model depends crucially on the concept that the cessation of proliferation and entry into meiotic arrest is controlled by the stage of development rather than by the size of the germ cell population. A more detailed analysis than the present report would be required to establish how robust the proposed mechanism may be to departures from each of these assumptions. There clearly are numerous models of greater complexity that could be proposed to account for the observed substantial variance among human oocyte populations. Our realization that the distribution of oocyte counts between individuals can be explained so simply by the computation described here encourages us to suggest that the small-founder effect may be a predominant cause of this variance.

Finally, we note that the squared root of an inversed moment ratio is mathematically equivalent to the coefficient of variation – the quotient of the standard deviation divided by the mean. This parameter is commonly used in biostatistics to characterize the extent of random variation across a broad range of biological processes. This coincidence suggests that the founder-effect interpretation that we have proposed may have broader applications.

## Competing interests

The author(s) declare that they have no competing interests.

## Authors' contributions

CJZ carried out the initial mathematical derivations and analyzed the oocytes-counting data using the moment-ratio method. EGL and SHM extended the mathematical derivations and completed the maximum-likelihood estimation. BB reviewed the biological implications of the models. All authors participated in preparations and revisions of the manuscript.
